# Double Digital Assay for Single Extracellular Vesicle and Single Molecule Detection

**DOI:** 10.1002/advs.202303619

**Published:** 2023-10-06

**Authors:** David E. Reynolds, Menghan Pan, Jingbo Yang, George Galanis, Yoon Ho Roh, Renee‐Tyler T. Morales, Shailesh Senthil Kumar, Su‐Jin Heo, Xiaowei Xu, Wei Guo, Jina Ko

**Affiliations:** ^1^ Department of Bioengineering University of Pennsylvania Philadelphia PA 19104 USA; ^2^ Department of Pathology and Laboratory Medicine University of Pennsylvania Philadelphia PA 19104 USA; ^3^ Department of Orthopaedic Surgery Perelman School of Medicine University of Pennsylvania Philadelphia PA 19104 USA; ^4^ Department of Biology School of Arts and Sciences University of Pennsylvania Philadelphia PA 19104 USA

**Keywords:** biomarker discovery, double digital microfluidics, extracellular vesicle, microwells, tyramide signal amplification

## Abstract

Extracellular vesicles (EVs) have emerged as a promising source of biomarkers for disease diagnosis. However, current diagnostic methods for EVs present formidable challenges, given the low expression levels of biomarkers carried by EV samples, as well as their complex physical and biological properties. Herein, a highly sensitive double digital assay is developed that allows for the absolute quantification of individual molecules from a single EV. Because the relative abundance of proteins is low for a single EV, tyramide signal amplification (TSA) is integrated to increase the fluorescent signal readout for evaluation. With the integrative microfluidic technology, the technology's ability to compartmentalize single EVs is successfully demonstrated, proving the technology's digital partitioning capacity. Then the device is applied to detect single PD‐L1 proteins from single EVs derived from a melanoma cell line and it is discovered that there are ≈2.7 molecules expressed per EV, demonstrating the applicability of the system for profiling important prognostic and diagnostic cancer biomarkers for therapy response, metastatic status, and tumor progression. The ability to accurately quantify protein molecules of rare abundance from individual EVs will shed light on the understanding of EV heterogeneity and discovery of EV subtypes as new biomarkers.

## Graphical abstract

This microfluidic technology demonstrates the ability to perform a double digital assay, allowing for the detection of single PD‐L1 protein molecules from individual EVs. The ability to accurately quantify rare protein molecules from individual EVs sheds light on the understanding of EV heterogeneity and the discovery of new EV subtype biomarkers.

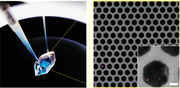

## 1 Introduction

Extracellular vesicles (EVs) are a group of heterogeneous lipid‐bound nanoparticles (30–200 nm exosomes, <1000 nm microvesicles, and >1000 nm apoptotic bodies) that are actively shed by cells in both healthy and pathological states.^[^ ^1^, ^2^ ^]^ Because EVs are involved in intracellular communication and exhibit high stability for protecting their molecular cargo (DNA, RNA, and proteins), they have emerged as promising diagnostic biomarkers for different types of cancers, infectious diseases, and neurological disorders.^[^ ^1^, ^2^, ^3^, ^4^, ^5^, ^6^, ^7^, ^8^ ^]^ However, the diagnostic application of EVs is challenged by its population heterogeneity and lack of sensitive detection methods. Therefore, new technologies and strategies are warranted for improving the accuracy and sensitivity measurement of EV cargo.^[^ ^1^, ^2^, ^9^, ^10^, ^11^ ^]^

In recent years, several advanced methods have been developed to resolve EV heterogeneity by detecting and characterizing individual extracellular vesicles (EVs). These technologies include single EV analysis (sEVA) using microscopic imaging,^[^ ^12^, ^13^, ^14^, ^15^, ^16^, ^17^, ^18^ ^]^ modified flow cytometry for EV analysis,^[^ ^19^, ^20^ ^]^ nano‐flow cytometry,^[^ ^21^, ^22^ ^]^ digital detection assays utilizing immunoaffinity capture (digital enzyme‐linked immunosorbent assay; dELISA) of EVs,^[^ ^6^, ^23^ ^]^ and nucleic acid‐based amplification.^[^ ^24^, ^25^ ^]^ Although these technologies have demonstrated remarkable success in profiling individual EVs, they are limited to bulk measurements of their molecules. In order to accurately parse out EV heterogeneity and discover different EV subtypes, it is necessary not only to profile single EVs, but also to accurately quantify their molecules. However, single EV analysis has already been challenging due to the extremely low molecular content at the single EV level, and it becomes even more complicated when attempting to count individual molecules from single EVs.

Digital assays are cutting‐edge techniques that allow for the precise counting of single target biomolecules (e.g., proteins, nucleic acids) or entities such as EVs or cells. These systems rely on the Poisson distribution (*λ* = 0.1) to prevent multiplets and digitally count analyte signals as single positive or negative events, achieving ultra‐sensitive detection.^[^ ^26^, ^27^ ^]^ While past studies have utilized multiple digital‐based technologies to detect and quantify the number of molecules of disease biomarkers in clinical samples,^[^ ^28^, ^29^, ^30^ ^]^ no current method exists that can combine EV digital assays with single‐molecule digital detection to achieve accurate biomarker expression levels at the single‐EV and single‐molecule resolution.

Herein, this paper presents a droplet‐free double digital assay that utilizes bead‐based microwell arrays and tyramide signal amplification (TSA) to achieve single EV, single molecule detection. The technique involves the use of microwells to compartmentalize individual EVs and form a monolayer of microbeads for single‐molecule capture and detection. By counting the number of fluorescent beads within the microwells, this platform can precisely profile the expression level of key EV biomarkers, improving our understanding of the composition of heterogeneous EV populations. The working principle of the technology began by demonstrating the microwell array's ability to partition a single EV per well. This was proven by loading and lysing A431 cell‐derived EVs in the device and characterizing their epidermal growth factor receptor (EGFR) protein with TSA. After single EV loading validation was confirmed, we subsequently used our method to assess the abundance of the programmed death‐ligand 1 (PD‐L1) protein in a melanoma cell line (624‐mel), proving the system's accuracy and sensitivity in detecting EV cargo for the identification of distinct EV subpopulations and biomarkers. Together, we believe the presented double digital technology provides a new way of EV biomarker characterization and discovery by enabling the absolute quantification of individual molecules from each EV.

## 2 Results and Discussion

### 2.1 Bead‐Based Digital Microwell Assays

**Figure 1:**
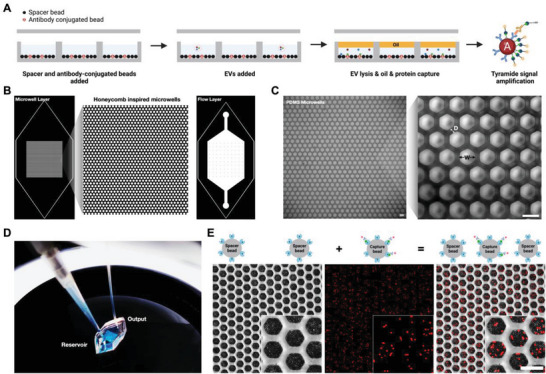
Device and schematic. A) Schematic of double digital EV microwell protein detection. B) CAD files of the bottom microwell layer and top flow layer. C) PDMS microwell devices: width (W) = 42 µm and distance (D) = 20 µm. (Scale bar 50 µm) D) Loading bonded microwell device with a solution in the reservoir and washing the system with a pump via connected tubing. E) Visualization of spacer streptavidin beads and capture streptavidin beads with conjugated biotin‐NHS‐Alexa Fluor (AF) 647 linker loaded into the microwell device. (Scale bar: 50 µm)

Digital microwell assays have emerged as powerful tools for compartmentalizing and profiling single cells. In particular, these devices have enabled the genomic, transcriptomic, and proteinic profiling of these single cells, opening the possibilities to new discoveries in biology and drug discovery.^[^ ^31^, ^32^, ^33^, ^34^, ^35^ ^]^ For most applications, these microwells are usually fabricated on a microfluidic device where they can be easily customized to accommodate different cell types.^[^ ^36^, ^37^ ^]^ Although their properties can be easily tuned, they have yet to be applied for single EV profiling. To address this challenge, we have developed a highly sensitive digital assay that allows for the partitioning of single EVs and quantification of their protein abundance. The general workflow for our technology begins with bead loading into microwells. Here, microbeads are used to capture individual protein molecules from single EVs, achieving digital ELISA. To resolve individual bead signals, a mixture of two types of beads is prepared where there are beads coated with capture‐targeting antibodies and other beads without antibody coating that serves as spacer beads. After bead loading, EV samples are loaded into the microwells, and lysis buffer is flowed into the device, immediately followed by oil to prevent cross‐contamination. After lysis, the TSA workflow is implemented for signal amplification. TSA is a technique for amplifying a signal that is weak or difficult to detect, allowing for the localization of the target molecule to a specific area.^[^ ^27^ ^]^ By depositing signal only at the site of amplification, TSA provides a localized signal that is contained to individual beads that can be easily recorded. (**Figure** **1**A) A more expanded workflow is provided in the supplementary information section. (Figure S1, Supporting Information)

Our technology was inspired by previously optimized microwell assays for single‐cell analysis. Several groups have fabricated a variety of different microwell designs, including circles, squares, honeycombs, and triangles.^[^ ^37^, ^38^, ^39^ ^]^ While many groups do not report many significant differences between these different shapes, honeycomb‐based designs remain popular for their handling, high seeding efficacy (high surface area‐to‐volume ratio), and uniform spacing between wells.^[^ ^40^, ^41^ ^]^ Therefore, we chose a honeycomb‐inspired design to achieve optimal bead loading with minimal bead loss. Our device is divided into two layers: bottom layer (microwells) and top layer (flow chamber). (Figure 1B) The bottom layer contains a lattice of 10 000 microwells and the top flow chamber contains a pillar array to prevent itself from collapsing onto the microwells. The two layers were fabricated with photo‐ and soft‐lithography to produce polydimethylsiloxane (PDMS) devices. (Figure 1C) After PDMS casting and curing, the cut and hole‐punched devices were then bonded together via plasma bonding to enclose the microwells. There are two aligned holes punched on opposite ends of the device: a reservoir for feeding solution into the device and a tubing connection port for washing the device with a pump. (Figure 1D) To determine whether single beads can be detected and counted in our bonded device, we loaded a cocktail of antibody‐coated beads and spacer beads into our system. We then added a fluorophore‐conjugated secondary antibody to stain and image antibody‐coated beads. During imaging, we observed that we can resolve and count individual fluorescent beads in each microwell. (Figure 1E) Using a software tool, we were able to segment and record individual fluorescent beads. (Figure S2, Supporting Information) Therefore, our technology served well for integrating microbeads into a microwell array and showed the feasibility of detecting individual molecules by counting the number of microbeads within a microwell.

### 2.2 Off‐Chip TSA Assay Validation

To detect scarce protein molecules from a single EV, we employed TSA. The TSA assay relies on the interaction between horseradish peroxidase (HRP) and tyramide. When tyramide reacts with HRP in the presence of hydrogen peroxide, phenolic groups in the tyramide become oxidized, producing tyramide radicals that form covalent bonds with aromatic amino acids rich in electrons. These radicals are then dispersed on the site of amplification, providing a localized signal that is contained to individual beads that can be easily recorded. In this case, the signal does not diffuse away, similar to ELISA. By labeling these tyramide radicals with fluorochrome or biotin, previously undetectable molecules can be observed. Thus, TSA serves well in its application for single EV molecular profiling, given their relatively low abundance of proteins.^[^ ^27^ ^]^ To demonstrate TSA's capacity for amplifying fluorescent signals, we targeted EGFR protein from both lysed cell and EV samples of a highly enriched EGFR protein‐based cell line (A431).^[^ ^42^ ^]^

**Figure 2:**
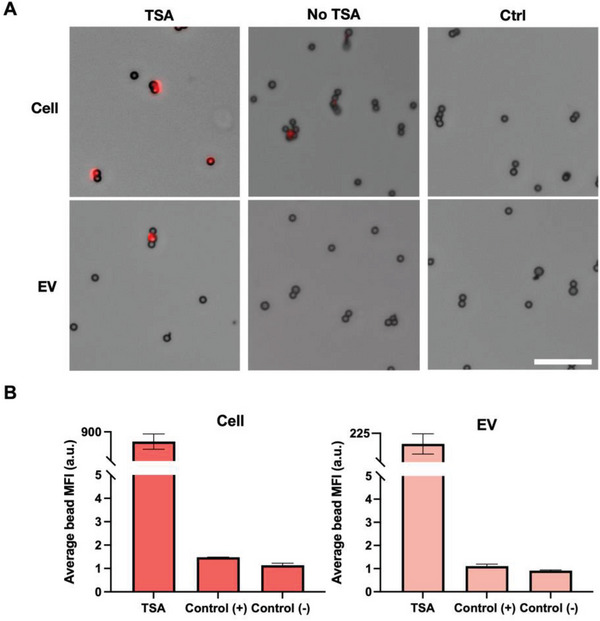
TSA off‐chip. A) EGFR protein capture and amplification from cell and EV lysis on epoxy beads: TSA, positive control (no TSA), and negative control (ctrl). (Scale bar = 20 µm) B) Quantification of mean fluorescence intensity (MFI = a.u.) from off‐chip TSA. For image analysis, the fluorescence intensity was measured from individual beads and then averaged among the others in the same frame. Three images were quantified for each condition (n=3).

Adapted from Yang et al.^[^ ^43^ ^]^ we applied their TSA workflow for our TSA off‐chip validation. Similarly, we implemented the assay on epoxy group coated beads, which facilitate the antibody coating process on‐bead. Because we were targeting EGFR protein from A431 cells and EV samples, an anti‐EGFR antibody was coated on the epoxy beads. Because EV lysis conditions remain unstandardized, we optimized the EV lysis conditions with Triton X‐100 and sonication. We uncovered no significant differences in the percentage of intact EVs with or without sonication and with 1% or 10% Triton X‐100 at 30 min of incubation. Thus, we chose 1% Triton X‐100 with no sonication. (Figure  S3, Supporting Information) By incubating the cell and EV lysate with the antibody‐coated beads, we were able to capture EGFR protein and amplify its detection with TSA successfully. (**Figure** **2**A) Comparatively, with no amplification, there were almost no fluorescent signals from EV samples. This is primarily attributed to EVs’ relatively low protein abundance compared to cells. Based on the fluorescence intensity, the TSA condition for the EV samples was over 100‐fold greater than both the unamplified and negative (no detection antibody) control samples. Similar trends were observed for the cell conditions based on the quantified fluorescence intensity. (Figure 2B) Therefore, the off‐chip validation proved the necessary integration of TSA to resolve undetectable EV protein signals.

### 2.3 Single EV loading and On‐Chip TSA

**Figure 3:**
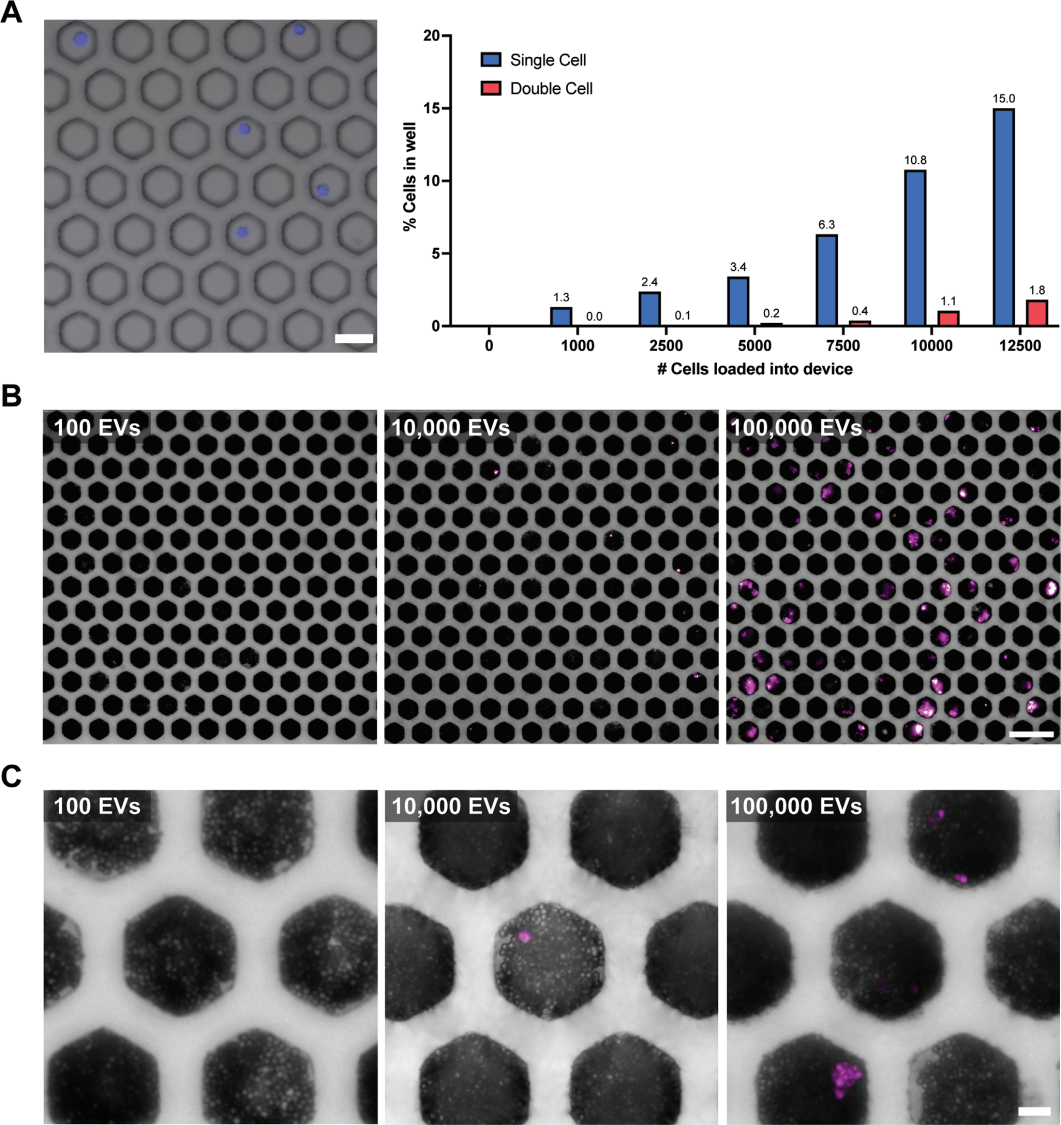
EV loading optimization for microwell array. A) Single‐cell loading into microwells at different concentrations. Expected vs actual single‐cell loading into individual microwells quantified. (Scale bar = 50 µm) For image analysis, individual fluorescent cells were counted from individual wells from three separate frames and then averaged. Three images were quantified for each condition (*n*=3). B) A431 EV titration into microwell array with EGFR protein capture and detection. (Scale bar = 100 µm). C) Magnified images A431 EV titration into microwell array with fluorescent EGFR protein captured beads and spacer beads. (Scale bar = 12.5 µm)

Single EV loading was optimized in our microwell array. In theory, because our device is occupied with 10 000 microwells, a diluted solution with 1000 EVs should be loaded into the device to achieve single entity loading. However, because our device is primarily occupied by dead space (an area without microwells), a series of titration experiments had to be performed to compensate for the loss of EVs. EVs, though, cannot be imaged in our microwell array, so cells were used to parametrize loading into our system. A431 cells, stained with Hoechst, were added at different cell numbers (0, 1000, 2500, 5000, 7500, 10 000, and 12 500) into the microwell array. To achieve digital loading (*λ* = 0.1), the Poisson distribution reports that 9.05% of the wells should be occupied by single entities. Based on imaging, we discovered that after 10 000 cells were added to our device, 10.8% of the wells were occupied with single cells. (**Figure** **3**A) In this case, to compensate for dead space loss, 10 000 entities must be loaded into the device to achieve the Poisson distribution range.

With the optimized single‐cell loading into our microwells, we then confirmed its translation with EVs. A431 EVs were loaded at different numbers (100, 1000, 10 000, 50 000, and 100 000 EVs) into the microwell array. These numbers were calculated based on NTA measurements after EV purification. Because single EVs cannot be imaged in our device and detected without amplification, we proceeded with our entire workflow to demonstrate its proof‐of‐principle. A cocktail of epoxy beads (EGFR capture antibody beads and spacer beads) was added to the device, followed by the EVs. After EV loading into the device, lysis, capture, and amplification were performed. The EVs were lysed to release the EGFR proteins throughout the microwell space for capture and detection on separate beads. To validate whether cross‐contamination occurs between wells, we loaded half our device with PBS and the other half with PBS suspended with 4 kD FITC‐dextran (300 µg mL^−1^). We then flowed oil over the wells to entrap the solutions. After 1 h, we did not observe FITC‐dextran bleed through into the other wells, confirming the efficacy of our system in safeguarding against cross‐contamination. (Figure S4, Supporting Information) Nonetheless, after conducting a series of titrations, we were able to verify that the concentration of 10 000 EVs yielded a number of fluorescent wells (≈10%) that closely matched the Poisson distribution range, as well as the reported single‐cell loading data. (Figure 3B) For the 100 000 EV‐loaded devices, based on the Poisson distribution (*λ* = 1), 40% of the wells are supposed to remain empty, which is demonstrated by this titration. Further analysis of both the cell and EV titrations is expanded in the Supporting Information (Figures S5 and S6, Supporting Information). With our highly sensitive digital ELISA platform, we demonstrate how we can perform single‐molecule digital detection (Figure 3C), as well as optimize for achieving single EV loading.

### 2.4 Single EV PD‐L1 Molecule Detection

After optimizing the TSA on‐chip assay with EGFR protein, we chose to apply our technology for the detection of important cancerous biomarkers, like PD‐L1. PD‐L1 is an immune checkpoint molecule that plays a significant role in immune evasion in cancer through its interaction with PD‐1. Numerous studies have demonstrated that the level of PD‐L1 positive(+) EVs in circulation significantly correlates with various aspects of tumor behavior, including size, metastatic status, and therapy response. This presents a new opportunity to develop PD‐L1(+) EVs as a cancer biomarker with promising prognostic and diagnostic potential.^[^ ^44^, ^45^, ^46^, ^47^ ^]^ However, there are several challenges associated with accurately and sensitively identifying PD‐L1(+) EVs. These include the limited availability of tumor‐derived EVs, their heterogeneous nature, and the presence of a significant background of EVs from diverse cell types.^[^ ^48^ ^]^ Because of these obstacles, their practical application in clinical settings has been suboptimal, leading to inconsistent outcomes. In this case, there is a pressing need to develop a technology that can accurately and comprehensively profile individual EVs and their PD‐L1 expression levels with a high degree of sensitivity. Thus, we applied our highly‐sensitive device to quantify the abundance and variability of PD‐L1 protein loading in single PD‐L1(+) EVs derived from a melanoma cell line (624‐mel).

**Figure 4:**
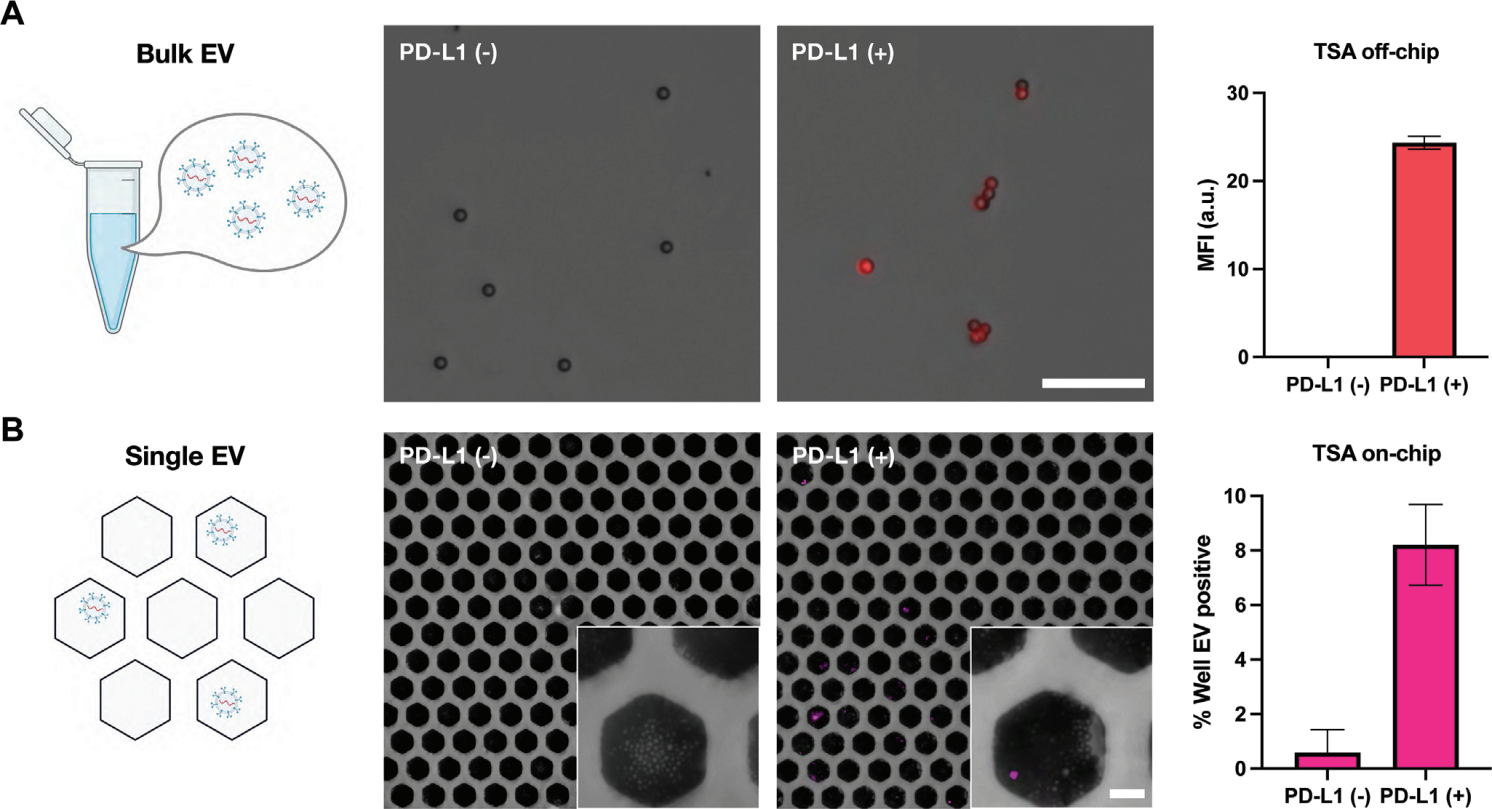
PD‐L1 protein detection in microwell array. A) Off‐chip PD‐L1 protein detection from PD‐L1(+/−) EVs. Quantification of mean fluorescence intensity (MFI = a.u.) from off‐chip TSA. (Scale bar = 20 µm) For image analysis, the fluorescence intensity was measured from individual beads and then averaged among the others in the same frame. Three images were quantified for each condition (*n*=3). B) On‐chip PD‐L1 protein detection from PD‐L1(+/‐) EVs. Quantification of wells positive for signal coming from single EVs. (Scale bar = 12.5 µm). For image analysis, individual fluorescent beads were counted from individual wells from five separate frames and then averaged. Five images were quantified for each condition (*n*=5).

To validate TSA's sensitivity and specificity, we detected PD‐L1 protein from bulk PD‐L1 expressing and PD‐L1 knockout 624‐mel derived EVs. The TSA off‐chip workflow was performed similarly to the EGFR off‐chip experiment, with the exception of anti‐PD‐L1 capture and detection antibodies. Based on the data, the PD‐L1(+) EV TSA condition generated a fluorescence signal, while the PD‐L1(−) TSA did not. Because of the EVs’ relatively low protein abundance, there was also no detectable signal for the sample without TSA as well. (Figure S7, Supporting Information) Through quantification, the fluorescence intensity for the PD‐L1(+) condition was over 20‐fold greater than the PD‐L1(−) condition. (**Figure** **4**A) This indicates the specificity of our TSA workflow and its application in amplifying PD‐L1 protein detection. We then transitioned to detecting PD‐L1 protein from single EVs in our microwell assay. With the optimized EV‐loading numbers reported in the previous section (10 000 EVs for *λ* = 0.1), we applied the same workflow for PD‐L1(+/−) EVs. Based on imaging, we confirmed that the PD‐L1(−) EV loading into the microwells did not generate a signal, while the PD‐L1(+) device did. Through quantification, we confirmed that ≈8% of our PD‐L1(+) microwell array had a signal, which is close to our previously reported single EV loading data. (Figure 4B) The relative differences in positive well signals can be attributed to differences in PD‐L1 expression between single EVs.

**Figure 5:**
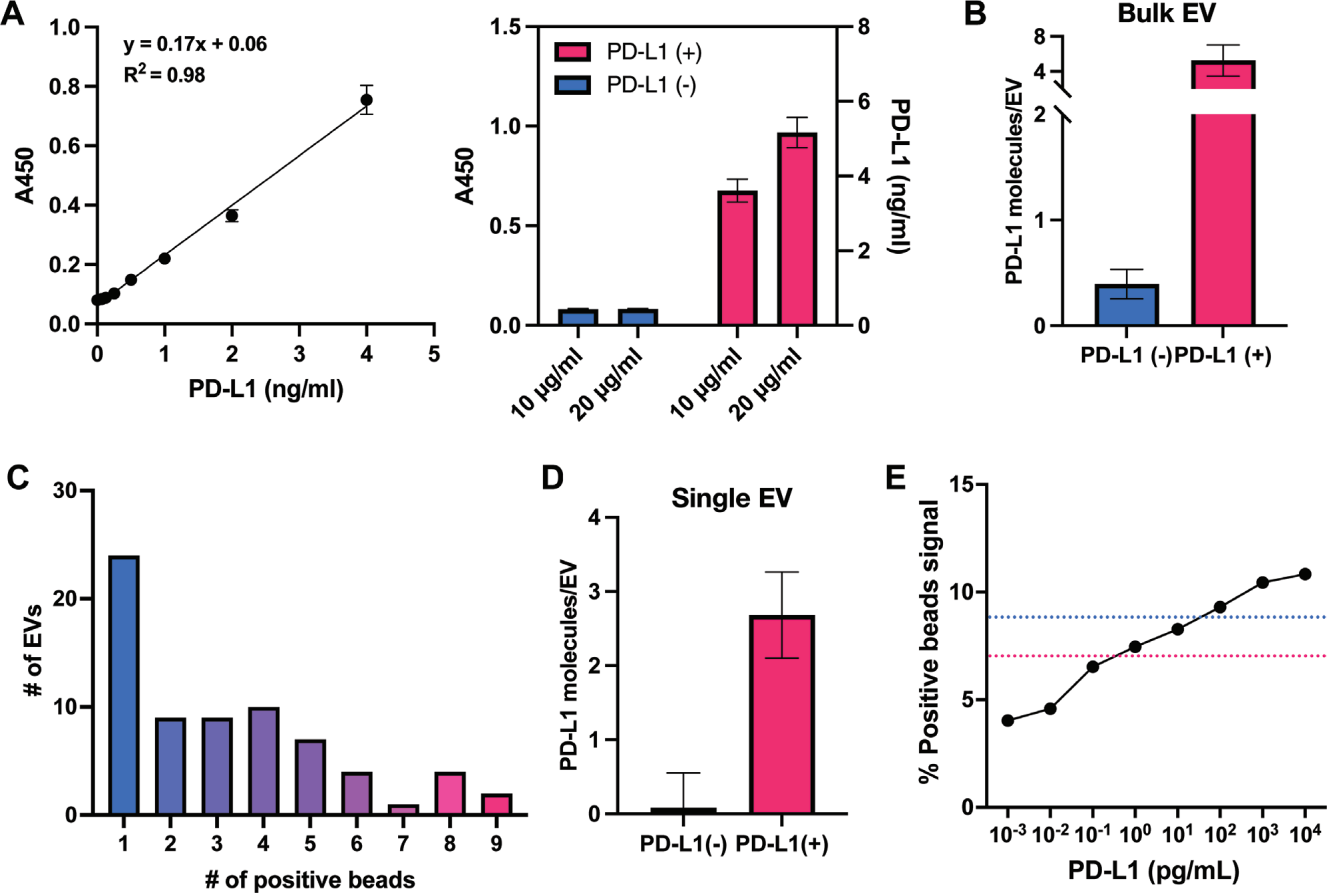
Digital counting of PD‐L1 molecules per EV. A) Standard curve created using recombinant human PD‐L1 proteins. The standard curve data presented in the figure were collected from triplicated individual samples (*n*=3). Bulk PD‐L1 (+/−) EVs loaded at different concentrations (10 and 20 µg mL^−1^) and detected with ELISA. B) Average number of PD‐L1 molecules coming from single PD‐L1(+/−) EVs based on ELISA. C) The number of positive beads per single PD‐L1(+) EV (*n*=70). D) The average number of PD‐L1 molecules per single PD‐L1(+/−) EV. E) The percent positive beads for synthetic PD‐L1 protein capture and detection. Three images were quantified for each condition (*n*=3). LOD reported for TSA (red) and ELISA (blue).

To verify the efficacy of our device in detecting single molecules, we conducted a series of off‐chip ELISA studies. First, a standard curve was made by serially diluting recombinant human PD‐L1 protein at different concentrations and detecting it with ELISA. Subsequently, PD‐L1 expression was measured using ELISA in PD‐L1(+/−) EVs at two different loading concentrations (10 and 20 µg mL^−1^). PD‐L1 concentration was calculated using the absorbance (A450) and the equation of the standard curve. Between the two EV concentrations, 10 µg mL^−1^ of PD‐L1(+) EV was found to have 3.65 ng mL^−1^ of PD‐L1 protein, which was within the range of the standard curve. (**Figure** **5**A) With this PD‐L1 concentration and known EV concentration (particles/mL) measured by NTA, the estimated number of PD‐L1 molecules per PD‐L1(+) EV was calculated to be between 4 and 6.5 molecules. (Figure 5B) This bulk EV measurement is limited to only providing an average number of PD‐L1 molecules expressed per EV. On the contrary, our double digital assay can profile individual EVs with their PD‐L1 expression. As expected, there was a high variability of PD‐L1 protein loading of 1–9 molecules per EV. (Figure 5C) On average, we detected 2.67 molecules EV^−1^ in PD‐L1(+) EV, comparable to the bulk EV measurement data. (Figure 5D) Example images of 1–9 molecules in a single well are provided in the supplementary information. (Figure S8, Supporting Information) Because our device's average fluorescent bead count is within the range of the ELISA, we can assume that our device is successfully detecting single molecules coming from single EVs.

To further demonstrate the necessary application of our system for single EV and molecule detection, we performed a series of fluorescent labeling (with anti‐PD‐L1 targeting antibodies) on bulk PD‐L1(+/−) EVs and imaged them with an inverted and super‐resolution fluorescence microscope. Based on imaging, no differences could be made between the PD‐L1(+) and PD‐L1(−) EVs, which indicates that PD‐L1 expression is extremely low at the single EV level and our double digital technology is needed to accurately profile key molecules from individual EVs. (Figure S9, Supporting Information)

To establish the limit of detection (LOD) for our system, we conducted a serial dilution (10^4^, 10^3^, 10^2^, 10^1^, 10^0^, 10^−1^, 10^−2^, and 10^−3^) using recombinant human PD‐L1 protein and antibody‐bound epoxy beads. Subsequently, we deployed the highly sensitive TSA method for signal detection. Our findings revealed a LOD of 0.4 pg mL^−1^, which is >60 times more sensitive than ELISA (LOD = 30 pg mL^−1^). (Figure 5E) The linear range appears to be between 0.1–1000 pg mL^−1^. It's noteworthy that our results concur with the existing reports, which describe an LOD ranging from 0.1–1 pg mL^−1^ for TSA.^[^ ^49^ ^]^ Altogether, we believe our findings corroborate our device's ability to detect single PD‐L1 molecules coming from single EVs, providing a highly sensitive, reliable, and robust platform for accurately profiling significant EV subpopulations and prognostic cancer markers.

## 3 Conclusion

The potential use of EVs as a source of biomarkers for disease diagnosis has generated significant interest. Despite their promise, the current diagnostic methods for EVs pose significant challenges. One of the primary issues is the low expression levels of biomarkers carried by EV samples, which make them difficult to detect accurately. Additionally, the physical and biological properties of EVs are complex, further complicating the diagnostic process. As a result, there is a pressing need to develop accurate and sensitive techniques that can overcome these challenges and unlock the full potential of EVs as a source of biomarkers. Thus, we have developed a double digital ELISA‐based assay that enables the absolute quantification of individual molecules from a single EV. Our approach involves the use of microwells to compartmentalize antibody‐coated and uncoated beads evenly, creating a monolayer that is ideal for subsequent digital ELISA. By counting the fluorescent beads within the microwells, our platform enables the precise determination of the expression level of specific biomarkers. With our device's ability to accurately quantify protein abundance and detect biomarkers, our system represents a promising step forward in the diagnosis and treatment of diseases.

We employed our integrative microfluidic technology to successfully measure protein abundance coming from single EVs. More specifically, we demonstrated how our technology can measure EGFR molecules coming from single EVs, demonstrating the efficacy of our technique. Not to mention, we demonstrated our ability to achieve the digital loading range for single EVs, which has yet to be demonstrated in microwells. Furthermore, we applied our device to measure the relative abundance of the PD‐L1 protein from a melanoma cell line. Based on our research, we have successfully demonstrated the ability of our device to capture and detect individual PD‐L1 molecules. Our chip yielded an average of 2.67 fluorescence beads per single EV‐positive PD‐L1(+) well, while our ELISA data revealed an average of 4–6.5 PD‐L1 molecules per individual PD‐L1(+) EV. This indicates that our technology is capable of detecting single molecules from single EVs, given that our device is relatively close to the detection threshold. Furthermore, our technology's sensitivity was confirmed by our LOD (0.4 pg mL^−1^), which is 60‐fold greater than ELISA. Altogether, we believe we have achieved a significant milestone, demonstrating the first‐ever double digital detection of single‐EV and single‐molecule.

While our technology has many advantages, there remain several limitations. For one, because TSA was used for amplification, the dependency of biotin and streptavidin in the assay prevents the usage of more user‐friendly beads like streptavidin‐coated beads. While epoxy beads serve well for our application, the conjugated require 20+ h incubation compared to 30 min. Not to mention, the TSA's chemistry inhibits the ability to target 4+ markers. In this case, further assay improvements would replace TSA with other amplification tools like rolling circle amplification to achieve higher multiplexing capacity. Another disadvantage to our system is the implementation of beads. While beads serve well for localizing and counting signals, they can become a burden when attempting to achieve and maintain a signal monolayer during loading and washing, respectively. In the future, we aim to circumvent the usage of beads in our device and rely on antibody‐coated surfaces. Although our system has its limitations, we believe the novelty in our approach for producing a double digital assay for single‐EV and single‐molecule detection, represents a promising step forward in the diagnosis and treatment of diseases.

## 4 Experimental Section

### Microwell and Top‐Flow Chamber Fabrication

The mylar photomasks were designed in AutoCAD and produced through Fineline Imaging. The silicon molds were fabricated at the Singh Center for Nanotechnology at the University of Pennsylvania. The microwell (*h* = 50 µm) and top flow chamber (*h* = 100 µm) layers were fabricated with soft lithography using SU‐8 3050. The PDMS devices were then bonded together via plasma bonding for 20 s at high power. To increase the hydrophobicity and remove pockets of air bubbles in the microwells, the bonded devices were incubated with Pluronic F‐127 (w/v: 0.05%) and degassed for 1 h at RT, respectively. The devices were then stored in Pluronic F‐127 at 4 °C until usage.

### Cell Culture and EV Isolation

A431 cells were grown in a 150 mm cell culture dish and then expanded to 12 dishes for EV isolation. DMEM (10% FBS, 1% penicillin) was used to culture and passage the cells. After the cells reached confluency, the medium was changed to exosome‐depleted DMEM (5% exosome‐depleted FBS, 1% penicillin). After 48 h from media exchange, the collected supernatant was spun at 400 g for 5 min and filtered with a 0.22 µm vacuum filter to remove cellular debris. The supernatant was centrifuged twice (Beckman Coulter) at 100 000 g for 70 min at 4 °C. The EV pellet was resuspended in PBS, aliquoted, and kept at −80 °C. PD‐L1 (+) and PD‐L1 (−) EVs were donated from the Wei Group.

### EV Characterization (Qubit, NTA)

Two different techniques were used to characterize the EVs. Qubit (Thermo Fisher) was used to assess the protein content, and nanoparticle tracking analysis (NTA) was used to determine how many particles were present. Thermo Fisher's protein assay kit was used for Qubit, and measurement was done in accordance with the manufacturer's instructions. The measurement for NTA was carried out at the University of Pennsylvania School of Veterinary Medicine Extracellular Vesicle Core (Zeta View by Particle Metrix). The analysis employed identical parameters (sensitivity of 75 and shutter of 75).

### PD‐L1 Antibody

To produce anti‐PDL1 antibodies (Gen Script), synthetic extracellular part (ECD) peptides of PD‐L1 were used to immunize mice. Standard ELISA was employed to test different clones of anti‐PDL1 antibodies for their reactivity to the PD‐L1 protein. At least 70 antibody clones were screened, from which pair‐matched clones 6G8 and 3F9‐Biotin were finally selected for the exosomal PD‐L1 ELISA assay. Anti‐PDL1 antibodies were donated from the Wei Group.

### Antibody Biotinylation

BSA‐free antibodies, secondary antibody AF647 (Thermo Fisher; A32787) and cetuximab (anti‐EGFR antibody, Selleckchem; A2000) were buffer exchanged to bicarbonate buffer (pH 8.4) using a 40k Zeba column (Thermo Fisher, 87 765). The antibody was then incubated for 30 min at room temperature (RT) with 20 molar equivalents of biotin‐NHS ester (Click Chemistry Tools; B102‐1G). Excess biotin‐NHS ester linker was then removed using a 40k Zeba column twice. The biotinylated antibodies were then stored at 4 °C until usage.

### Bead Loading Optimization

Streptavidin‐coated magnetic beads (Spherotech; SVM‐40‐10) were used for bead loading optimization. Before the beads were loaded, they were washed with D.I. water on a PCR magnetic tube rack. To create biotin‐NHS‐AF647 labeled streptavidin‐coated beads, the biotin‐NHS‐AF647 conjugate was incubated with the streptavidin‐coated beads in bicarbonate buffer (pH 8.4) for 30 min at RT on a rocker. After incubation, unbound NHS‐647F was washed away with PBS. A cocktail of biotin‐NHS‐AF647 labeled and spacer streptavidin‐coated beads were then loaded into the device. The beads were allowed to settle for an hour at RT and then centrifuged at 100 × *g* for 1 min to encourage monolayer displacement of the beads. The device was then imaged using an Olympus IX83 inverted fluorescence microscope.

### Epoxy Bead Coating

Epoxy magnetic beads (Spherotech; EM‐20‐10) were prepared for antibody conjugation. The magnetic beads were washed with D.I. water on a PCR magnetic tube rack. For every 1.6E6 of epoxy beads, 1.25 µg of anti‐EGFR and anti‐PD‐L1 (6G8) antibodies were prepared. The beads and antibody were resuspended into 200 µL of carbonate buffer (pH 9.0), and incubated on a rocker for 20 h at 37 °C. After incubation, the beads were washed and resuspended in PBS. The antibody‐bound epoxy beads were then stored at 4 °C until usage.

### EGFR and Pd‐L1 TSA Detection Off‐Chip

For A431 cell lysate, 1.0E6 fresh cells were pelleted and incubated with a 1X working concentration of protease inhibitor cocktail (Thermo Fisher; 78 430) in 1 mL of RIPA lysis and extraction buffer (Thermo Fisher; 89 900) on ice for 15 min. After incubation, the solution was centrifuged at 14 000 g for 15 min to pellet the cell debris. The supernatant was then transferred, and its protein concentration was assessed with Qubit. Protein was then stored at −80 °C until usage. For A431 and 624‐mel EV lysate, EVs were incubated with 1% Triton X‐100 at RT for 30 min. Once the lysis was complete, the protein was quantified with Qubit.

For the TSA protocol, all incubations were performed on a rocker at RT, and all wash steps were performed on a PCR magnetic rack. First, the antibody‐bound epoxy beads were incubated with a blocking buffer (2% BSA‐PBS) for 30 min. After blocking, the beads were washed three times with a wash buffer (PBS + 0.1% Tween‐20). The beads were then incubated with cell, or EV lysate in the blocking buffer for 1 h. For every 1.6E6 beads (antibody‐bound epoxy bead), 250 ng of cell, or EV protein was prepared. After protein capture, the beads were washed three times with the wash buffer. The biotinylated detection antibody (anti‐EGFR or anti‐PD‐L1 (3F9)) was resuspended in blocking buffer at a concentration of 0.5 µg mL^−1^ and incubated with the beads for 1 h. The beads were subsequently washed three times with the wash buffer. The beads were then incubated with the streptavidin‐HRP (Thermo Fisher; 21 130), diluted in 137.5 ng mL^−1^ of blocking buffer + 0.1% Tween‐20, for 30 min. After incubation, the beads were then washed three times with the wash buffer. The beads were then incubated with biotin tyramide (Sigma; SML2135) for signal amplification, diluted at a concentration of 0.5 µg mL^−1^ in 0.1 m borate buffer (pH 8.5) + 0.003% H_2_O_2_, for 10 min. Once amplification was complete, the beads were washed and then incubated with streptavidin‐647 fluorophore (Biolegend; 405 237), diluted in 0.5 µg mL^−1^ of blocking buffer, for 30 min. Finally, the beads were washed and imaged.

### LOD for TSA PD‐L1 Detection

Recombinant human PD‐L1/B7‐H1 protein (R&D; 156‐B7) was used for validating the LOD of TSA. Using anti‐PD‐L1 (6G8) antibody‐bound epoxy beads, a series of different concentrations of recombinant PD‐L1/B7‐H1 protein was incubated with the beads. The same protocol and reagents as the TSA detection off‐chip with PD‐L1 (+/−) EVs were applied in this workflow.

### PD‐L1 Detection with ELISA

ELISA assay was performed in 96‐well plates (Corning High Bind Microplate; 9018) according to the manufacturer's instructions. Briefly, the plates were coated with 50 µL of capture antibody, the anti‐human PDL1 monoclonal antibody, clone 6G8, at a concentration of 5 µg mL^−1^ in PBS and incubated overnight at 4 °C. After washing the wells five times with PBS containing 0.05% Tween‐20 (PBST), 200 µL of blocking buffer (1% BSA in PBST) was added to each well, and the plate was incubated for 1 h at RT.

Purified 624mel cell line‐derived EVs were diluted in PBS to different concentrations, and 100 µL of each dilution was added to the wells in triplicate. The plate was incubated overnight at 4 °C with gentle shaking and then washed five times with PBST. Biotinylated anti‐human PD‐L1 monoclonal antibody, clone 3F9, was added at a concentration of 1 µg mL^−1^, and the plate was incubated for 1 h at RT. After washing the wells five times with PBST, streptavidin‐HRP (BD Bioscience) was added and incubated for 1 h at RT.

Finally, the plate was washed five times with PBST and developed with 100 µL of TMB substrate solution. The reaction was stopped with 100 µL of H_2_SO_4_, and the absorbance was measured at 450 nm using a microplate reader (BioTek). A standard curve was generated using recombinant human PD‐L1 protein (R&D Systems; 156‐B7) at concentrations ranging from 0.06–4 ng mL^−1^. The PD‐L1 concentration in the samples was calculated based on the standard curve. The number of PD‐L1 per EV was calculated based on the molecular weight of PD‐L1 ranging from 33–55 kD.

All incubations and washes were performed using an automated plate washer (Fisher Scientific). The data were analyzed using GraphPad Prism software.

### EGFR and PD‐L1 TSA Detection On‐Chip

For each incubation step, the devices were left at RT without agitation or movement. And, for all the wash steps, the devices were washed with 60 µL of wash buffer at a flow rate of 30 µL h^−1^. For TSA on‐chip, a cocktail of antibody‐coated and spacer epoxy beads were loaded into the microfluidic device. After 1 h of bead incubation in the device, the device was centrifuged at 100 × *g* for 1 min to create a single monolayer of beads in each well. The device was then loaded with EVs and were allowed to settle for an hour. Subsequently, the device is flowed in with lysis buffer (1% Triton X‐100) and then immediately oil (Fluo‐oil 7500) to prevent cross‐contamination. The device was then incubated with the two‐phase solution system for 1.2 h. After lysis, the detection antibody, followed by the streptavidin‐HRP, biotin tyramide, and streptavidin‐647 fluorophore were all incubated with the device with the same off‐chip protocol and imaged.

### Super‐Resolution Imaging of EVS

AF488‐NHS (Sigma; 41698‐1MG‐F) was first used to stain all EVs by targeting the surface protein of PD‐L1 EVs. EVs (3.4 µg) were mixed with 6 µL of AF488‐NHS (1 mm), and the reactions were brought to a final volume of 12 µL with bicarbonate buffer (pH 8.4). After 2 h of reaction at RT, excess AF488‐NHS was removed using a 40k Zeba column twice. Then, stained EVs were introduced to an 8‐well‐chambered cover glass (Cellvis, CA, USA) and incubated for 30 min to deposit EVs on its surface. The cover glass was then washed with 1x PBS and blocked for 30 min by using 1% BSA. Then, 5 µg mL^−1^ of primary PD‐L1 antibody (3F9‐biotin) diluted in 1% BSA was incubated with the sample at RT for 1 h. After unbound antibodies were washed away, 2 µg mL^−1^ of AF647‐labeled secondary antibodies (strep‐AF647) were incubated with the cover glass for 1 h at RT. Finally, samples were washed with 1× PBS.

After exchanging 1x PBS for imaging buffer (10% glucose, 100 mm Cysteamine, 1% GLOX in PBS),^[^ ^50^ ^]^ dSTORM imaging was acquired on an ONI nanoimager (Oxford Nanoimaging, Oxford, UK) equipped with 405, 488, 561, and 640 nm lasers. Two‐channel dSTROM data was acquired using the 488 and 640 nm laser with a power of 200 and 400 mW, respectively, and an exposure time of 20 ms with 1500 frames. Data were drift‐corrected and filtered using CODI software (Oxford Nanoimaging) to minimize low‐precision and non‐specific localization. A diameter between 30 and 240 nm and circularity >0.3 were considered EVs. Individual EV clusters and the number of counts were analyzed using CODI software. Note that the number of counts from PD‐L1 was taken from dual positive EVs.

### EV Lysis

EV labeling was performed with Calcein Green AM (Biolegend; 425 201). EV and Calcein Green AM (1 mM) aliquots were thawed at room temperature. The Calcein Green AM stock aliquot was diluted to 10 µM in PBS with 1.0E7–1.0E8 EVs. The working solution was then incubated at 37 °C for 20 min. After incubation, the EVs were aliquoted into their separate concentrations of Triton X‐100. For sonication, the EVs were sonicated for 30 min in a Branson 3500 sonicator instrument at 40 kHz, while the non‐sonicated EVs were left in the lysis buffer at RT for 30 min. After incubation, the EVs were pipetted onto individual wells of a PTFE‐printed slide at 20 µL and allowed to settle on the glass for 20 min. The wells were then washed three times with PBS and immediately imaged.

### Statistics

Statistical analyses and line fitting were performed in GraphPad Prism 10. Data were used “as is” and was not pre‐processed.All data were normalized against positive controls, and no outliers were excluded. Data were presented as mean ± standard deviation for off‐chip TSA and PD‐L1 studies. Sample sizes (*n*) are included in figure captions. FIJI (ImageJ) software was used for all fluorescence quantification and image preparation. For bead counting, fluorescent beads were segmented and counted using a Python computer vision program with a CV2 plugin.

## Conflict of Interest

The authors declare no conflict of interest.

## Supporting information

Supporting Information

## Data Availability

The data that support the findings of this study are available from the corresponding author upon reasonable request.
