# Supplementary material for: Double Digital Assay for Single Extracellular Vesicle and Single Molecule Detection
Source: Adv Sci (Weinh). 2023 Oct 6;10(33):2303619. doi: 10.1002/advs.202303619 (PMC10667851; doi:10.1002/advs.202303619)
Supplement: Supplementary file 1 — Supporting Information [file ADVS-10-2303619-s001.pdf]

ADVANCED SCIENCE
Open Access

Supporting Information

for *Adv. Sci.*, DOI 10.1002/advs.202303619

Double Digital Assay for Single Extracellular Vesicle and Single Molecule Detection

*David E. Reynolds, Menghan Pan, Jingbo Yang, George Galanis, Yoon Ho Roh, Renee-Tyler T. Morales, Shailesh Senthil Kumar, Su-Jin Heo, Xiaowei Xu, Wei Guo and Jina Ko**

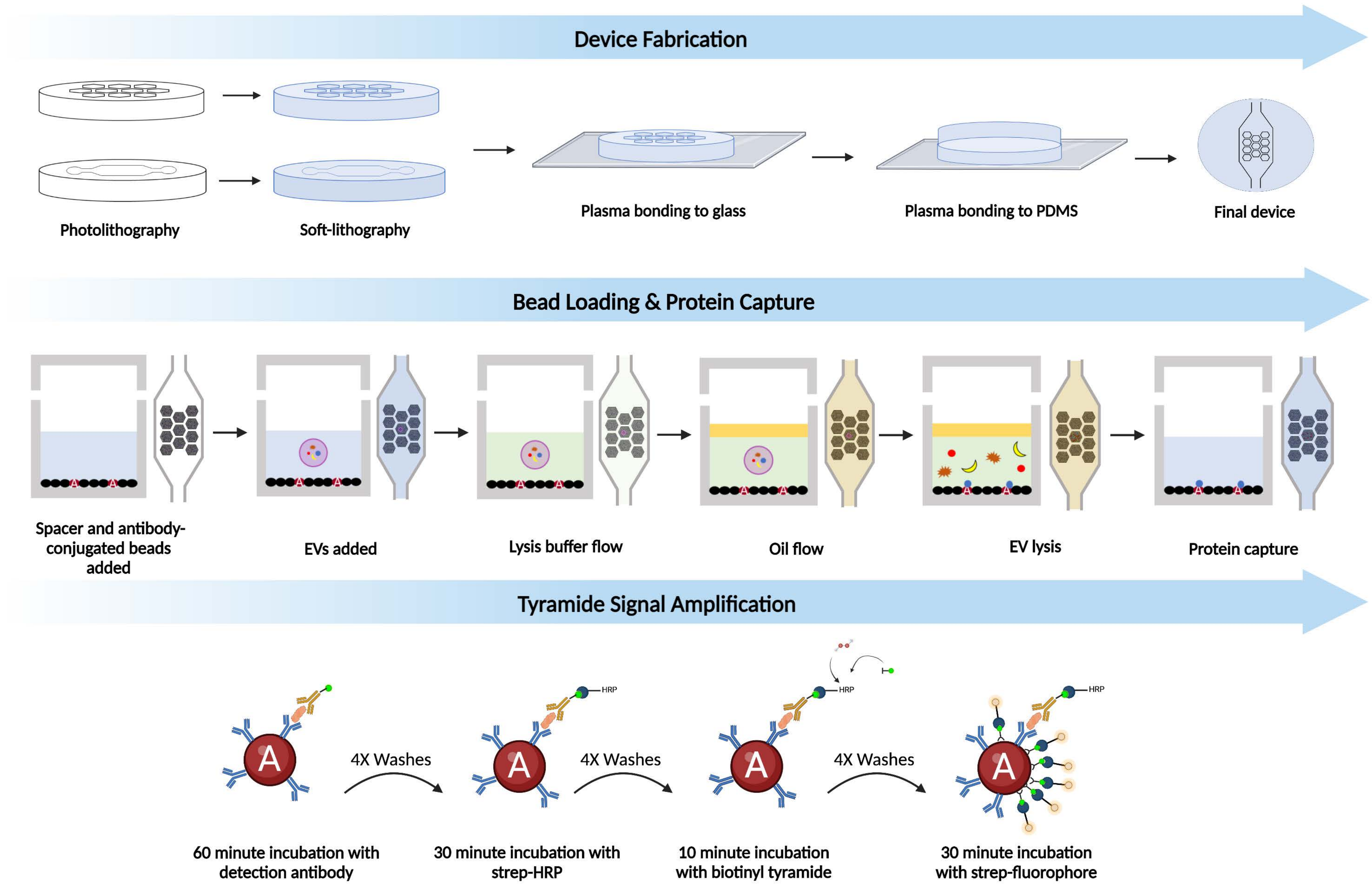

Supplementary Figure 1. Extended schematic for EV microwell assay.

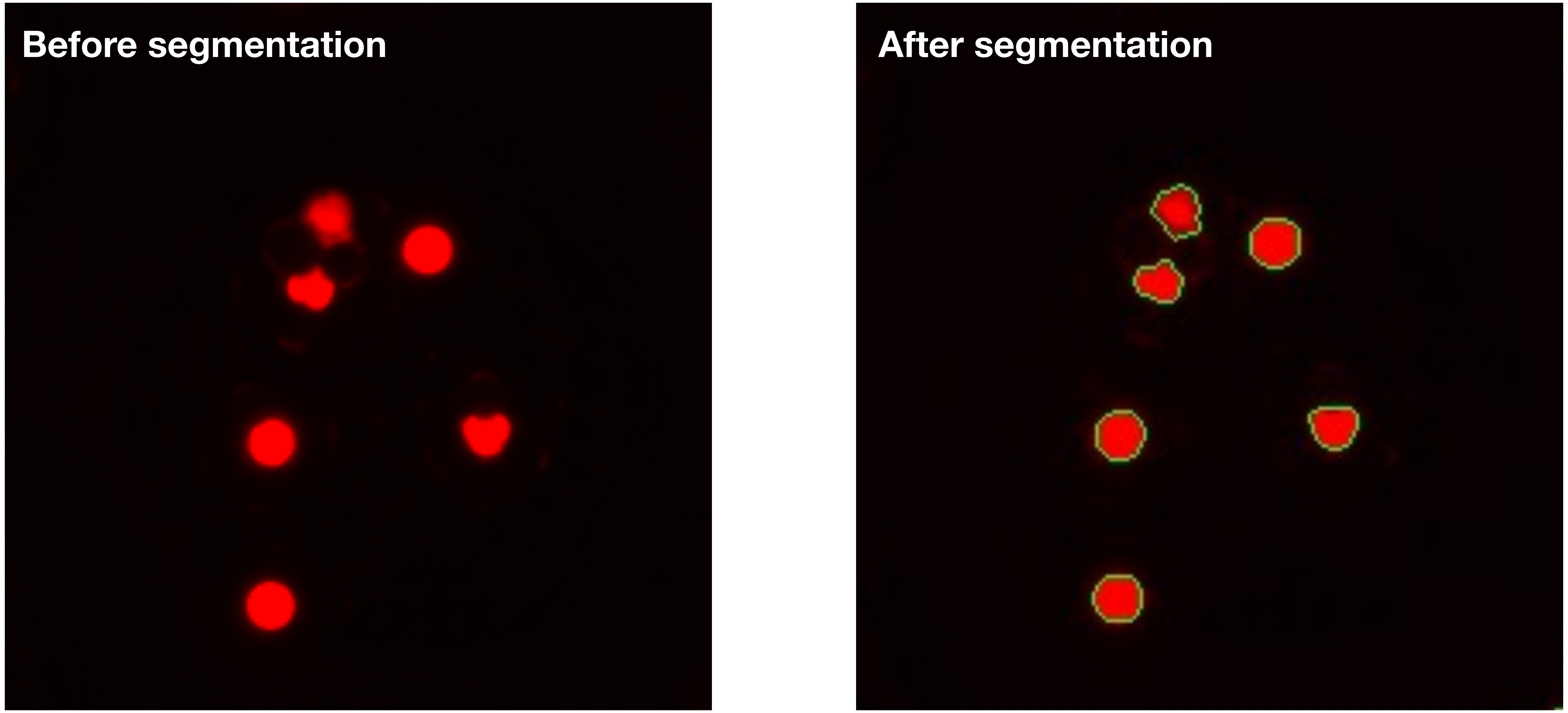

Supplementary Figure 2. Fluorescent beads are segmented and counted using Python computer vision program with a CV2 plugin.

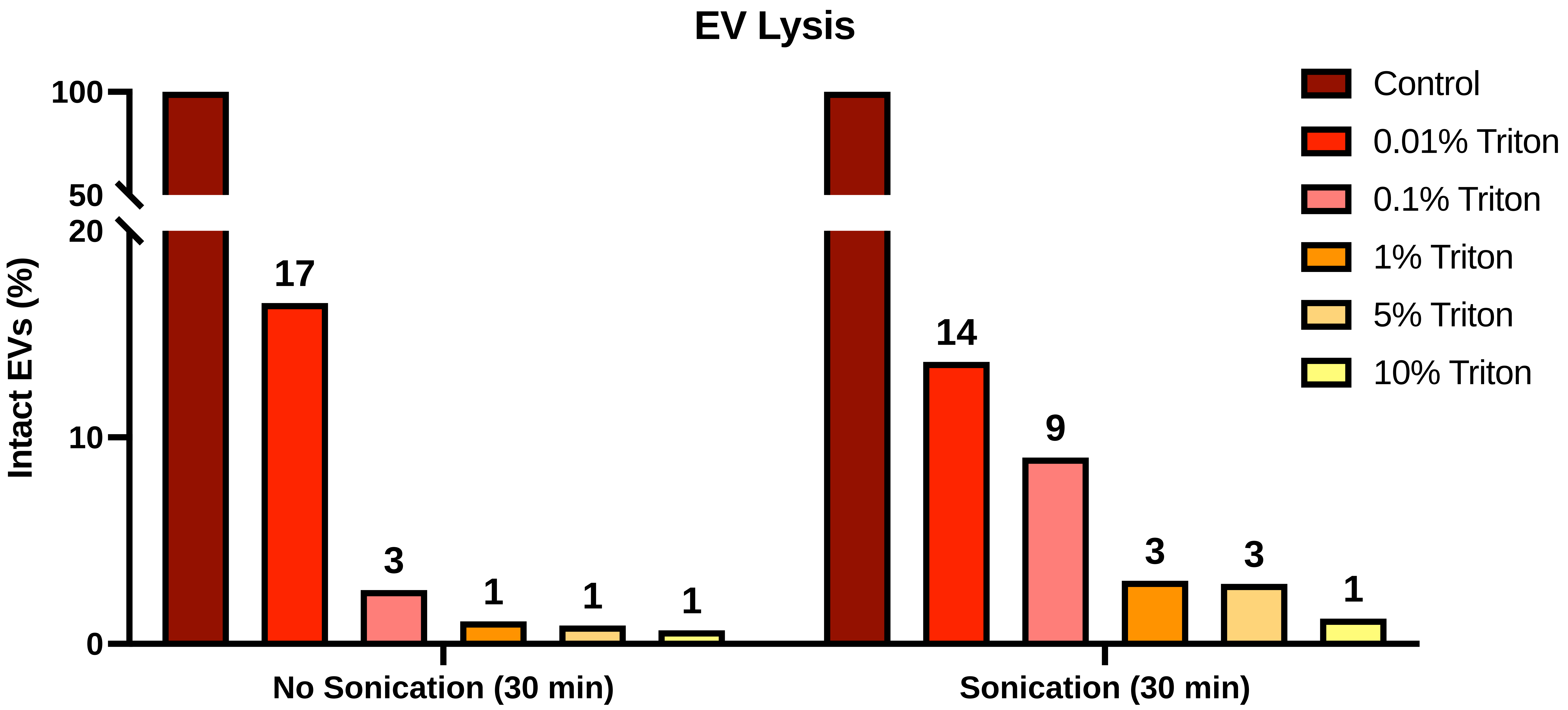

Supplementary Figure 3. EV Lysing optimization with different conditions: with/out sonication and Triton X-100. For image analysis, three images were quantified for each condition (n=3).

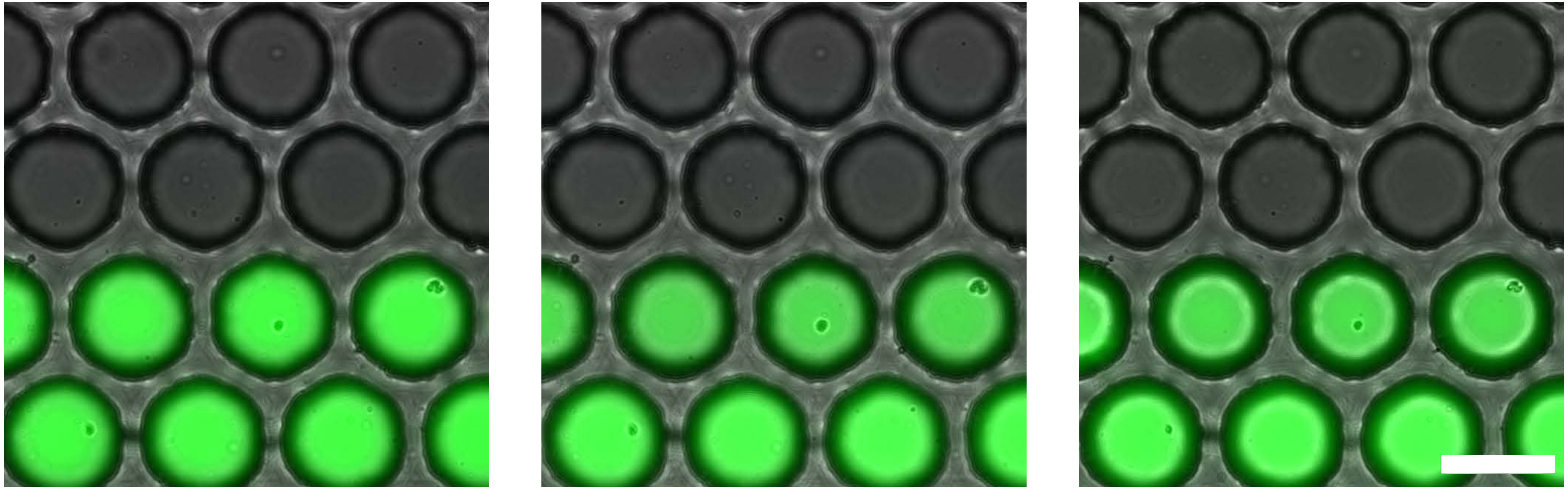

Supplementary Figure 4. Cross-contamination between PDMS microwells was verified with PBS suspended in FITC dextran and oil. Images were taken at three different time points (0, 30, and 60 minutes). (Scale bar = 50 μm)

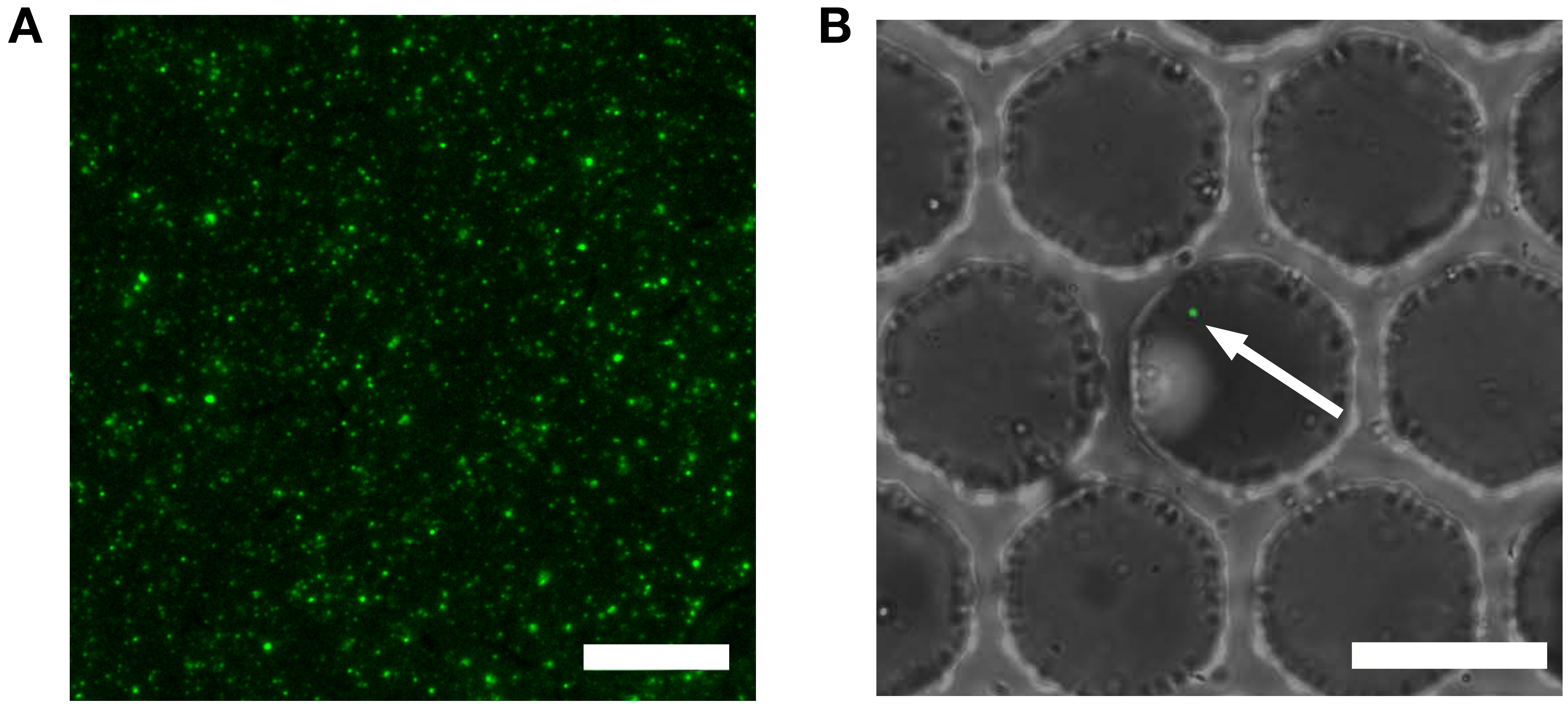

Bulk EVs **Single EV**

Supplementary Figure 5. EV calcein green AM staining. **A**) Bulk EV staining on a glass slide. (scale bar = 20 μm) **B**) Single EV loading into PDMS microwells. (scale bar = 50 μm).

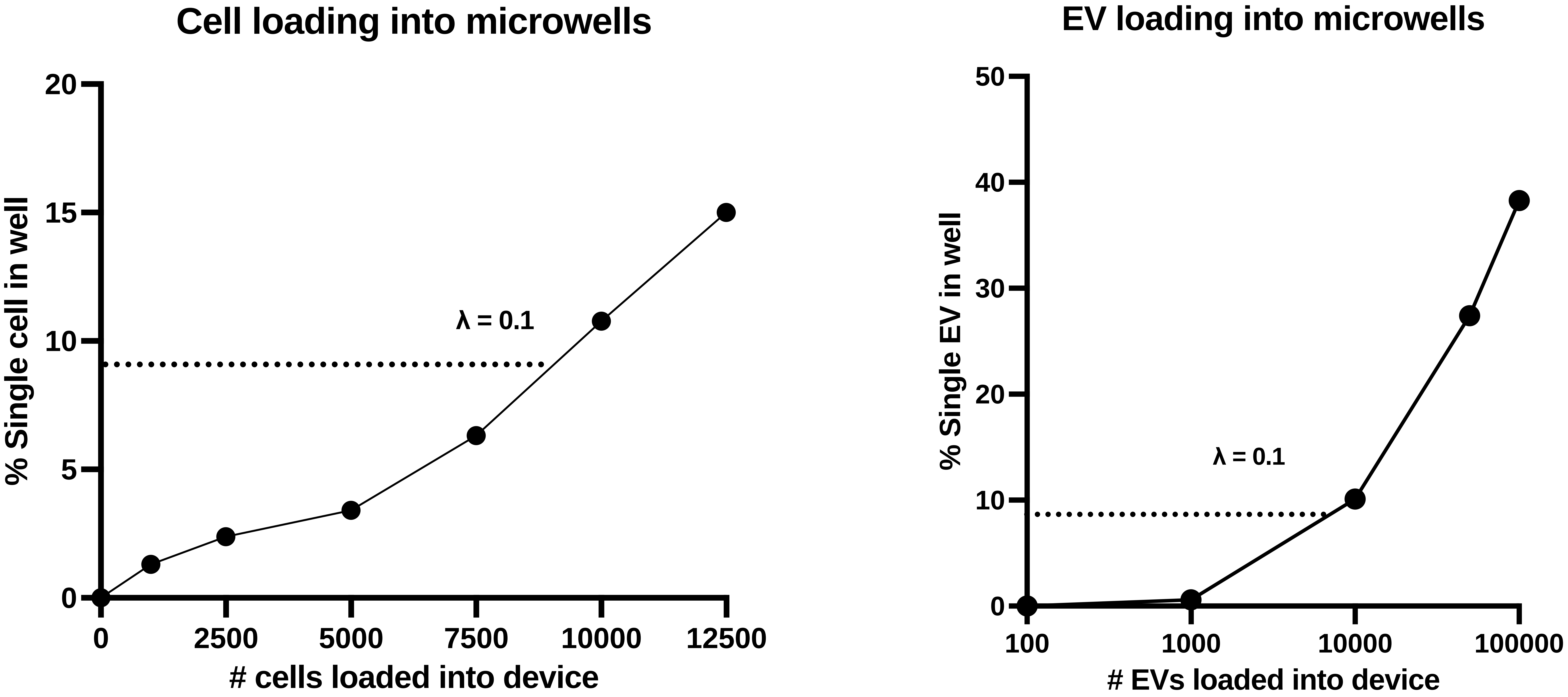

Supplementary Figure 6. Single-cell and EV loading into microwells. Lambda (λ) is reported on each graph. For image analysis, individual cells and fluorescent beads were counted from individual wells from 3 separate frames and then averaged (n=3).

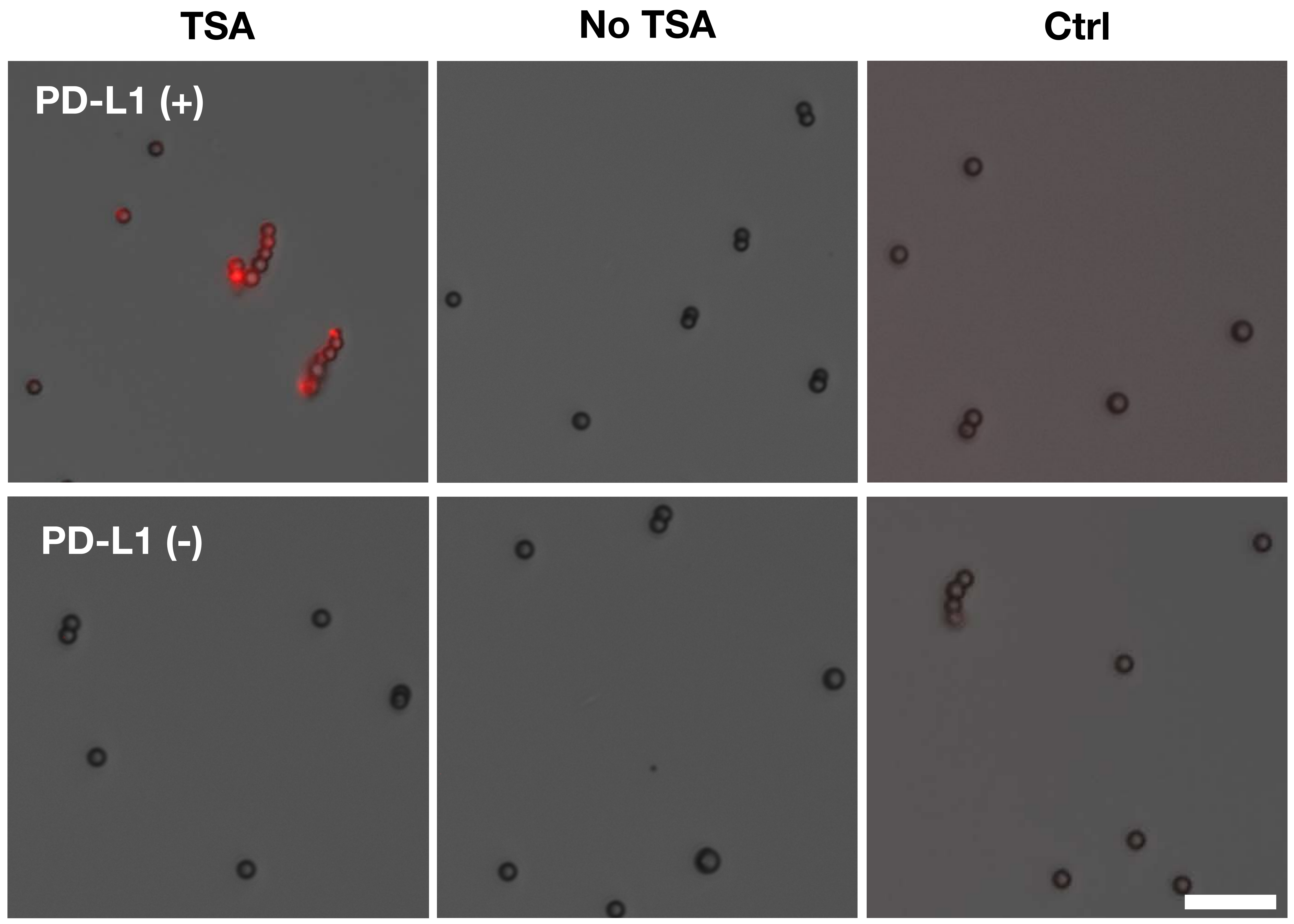

Supplementary Figure 7. PD-L1 protein detection off-chip. Images with TSA, positive control (no TSA), and negative control (ctrl). (Scale bar = 20 μm)

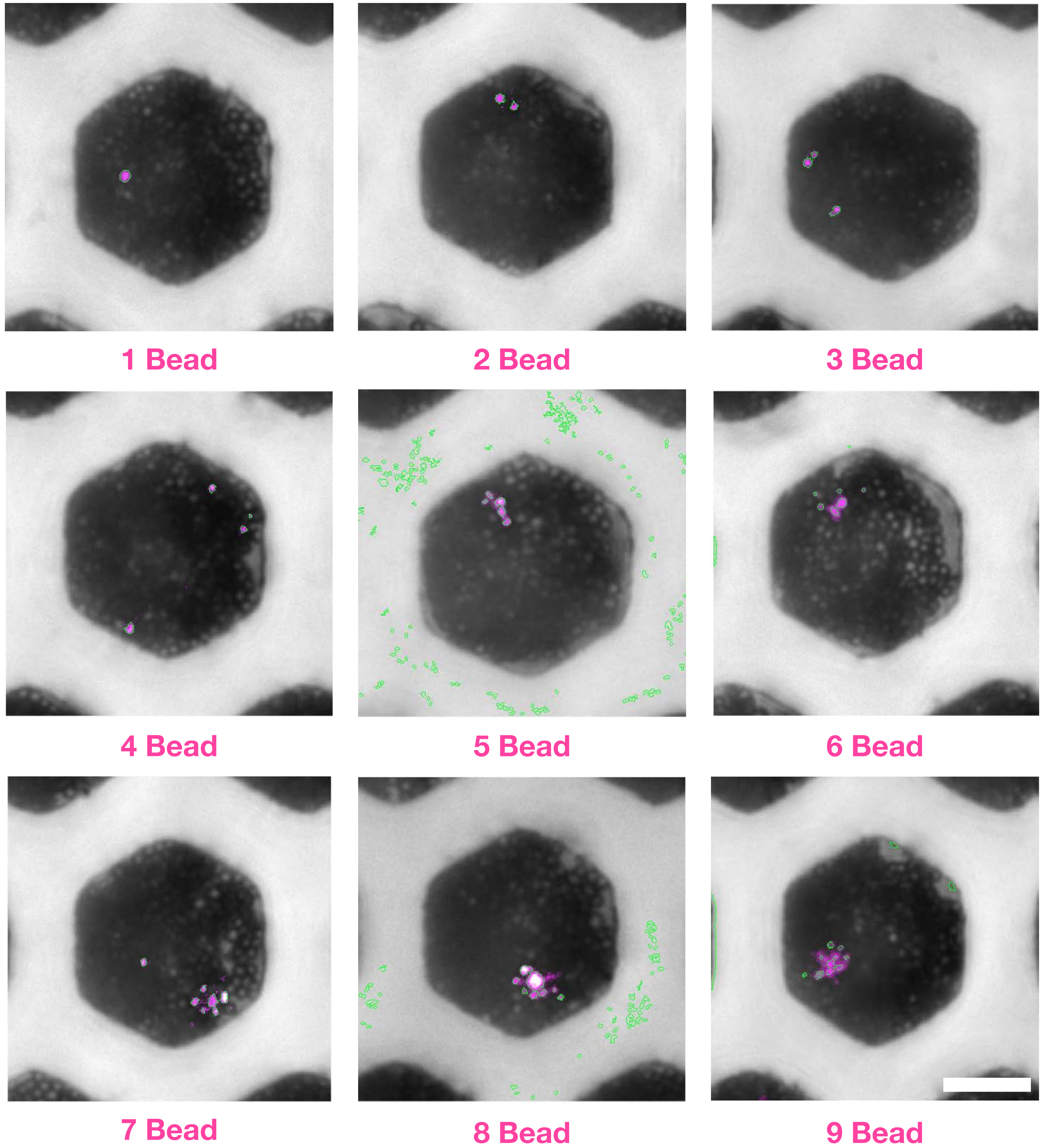

Supplementary Figure 8. Representative images showing individual positive fluorescent beads in individual microwells. (Scale bar = 20 μm)

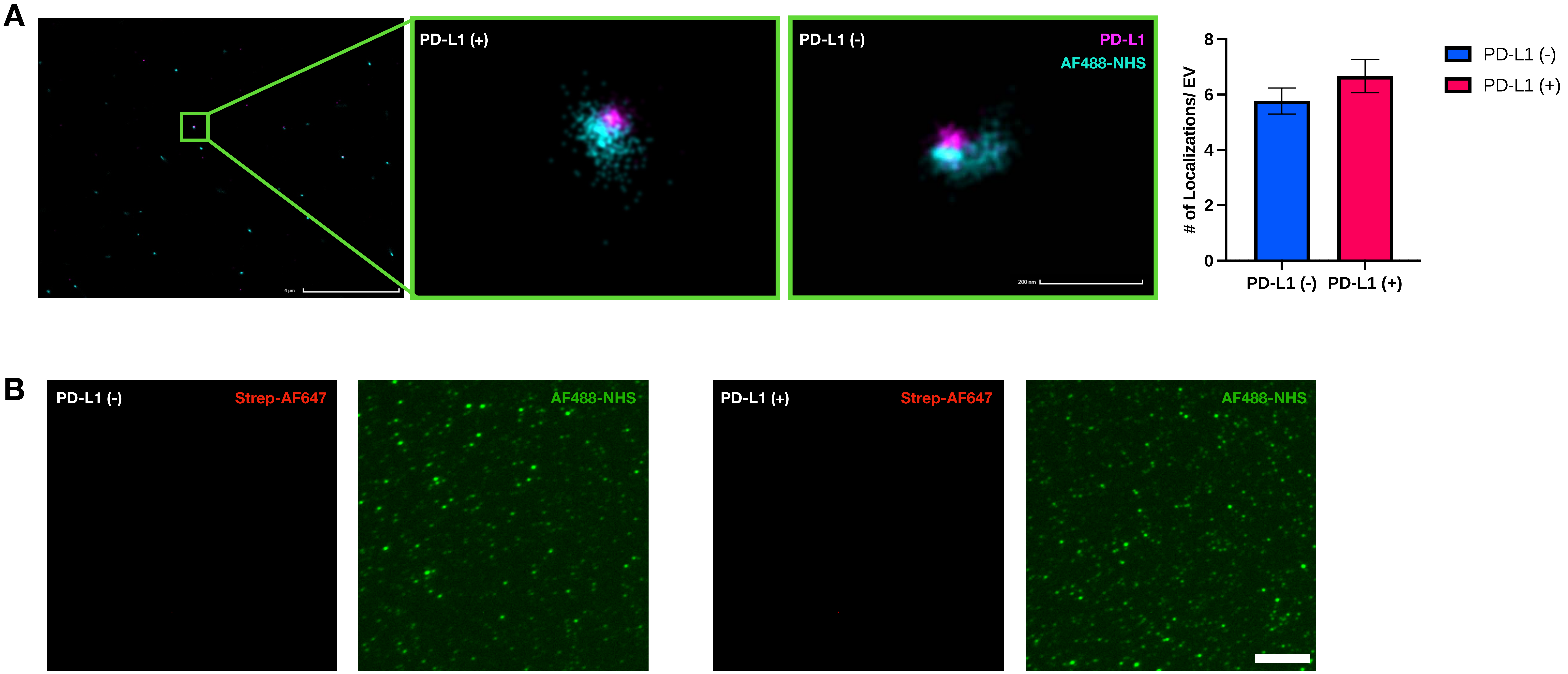

Supplementary Figure 9. Supplementary Figure 5. PD-L1 (+/-) EV fluorescence imaging. **A**) Super-resolution imaging of PD-L1 (+/-) EVs. The number of blinking events was counted from double-positive EVs (error bar = standard error). **B**) Fluorescence-based imaging of PD-L1 (+/-) EVs with an inverted fluorescence microscope.
